# Interaction between Calcium Chelators and the Activity of P2X7 Receptors in Mouse Motor Synapses

**DOI:** 10.3390/ijms21062034

**Published:** 2020-03-16

**Authors:** Anna Miteva, Alexander Gaydukov, Olga Balezina

**Affiliations:** Department of Human & Animal Physiology, Lomonosov Moscow State University, Leninskie Gory 1/12, Moscow 119991, Russia; anka.miteva@gmail.com (A.M.); balezina@mail.ru (O.B.)

**Keywords:** P2X7 receptors, L-type VDCCs, EGTA-AM, BAPTA-AM, neuromuscular junction

## Abstract

The ability of P2X7 receptors to potentiate rhythmically evoked acetylcholine (ACh) release through Ca^2+^ entry via P2X7 receptors and via L-type voltage-dependent Ca^2+^ channels (VDCCs) was compared by loading Ca^2+^ chelators into motor nerve terminals. Neuromuscular preparations of the diaphragms of wild-type (WT) mice and pannexin-1 knockout (Panx1^−/−^) mice, in which ACh release is potentiated by the disinhibition of the L-type VDCCs upon the activation of P2X7 receptors, were used. Miniature end-plate potentials (MEPPs) and evoked end-plate potentials (EPPs) were recorded when the motor terminals were loaded with slow or fast Ca^2+^ chelators (EGTA-AM or BAPTA-AM, respectively, 50 μM). In WT and Panx1^−/−^ mice, EGTA-AM did not change either spontaneous or evoked ACh release, while BAPTA-AM inhibited synaptic transmission by suppressing the quantal content of EPPs throughout the course of the short rhythmic train (50 Hz, 1 s). In the motor synapses of either WT or Panx1^−/−^ mice in the presence of BAPTA-AM, the activation of P2X7 receptors by BzATP (30 μM) returned the EPP quantal content to the control level. In the neuromuscular junctions (NMJs) of Panx1^−/−^ mice, EGTA-AM completely prevented the BzATP-induced increase in EPP quantal content. After Panx1^−/−^ NMJs were treated with BAPTA-AM, BzATP lost its ability to enhance the EPP quantal content to above the control level. Nitrendipine (1 μM), an inhibitor of L-type VDCCs, was unable to prevent this BzATP-induced enhancement of EPP quantal content to the control level. We propose that the activation of P2X7 receptors may provide additional Ca^2+^ entry into motor nerve terminals, which, independent of the modulation of L-type VDCC activity, can partially reduce the buffering capacity of Ca^2+^ chelators, thereby providing sufficient Ca^2+^ signals for ACh secretion at the control level. However, the activity of both Ca^2+^ chelators was sufficient to eliminate Ca^2+^ entry via L-type VDCCs activated by P2X7 receptors and increase the EPP quantal content in the NMJs of Panx1^−/−^ mice to above the control level.

## 1. Introduction

To date, several sources of Ca^2+^ influx into the nerve terminals of mammalian motor synapses have been described [1,2,3]. The main and most important source is calcium influx via P/Q-type voltage-dependent Ca^2+^ channels (VDCCs), which triggers the exocytosis of synaptic vesicles in the active zone [4]. In addition, L-type VDCCs that are in a silent state and usually do not affect Ca^2+^-dependent acetylcholine (ACh) release have been detected in motor nerve terminals. However, in the case of disinhibition, these channels play a role in effectively increasing the evoked release of ACh [5,6,7,8]. Finally, ionotropic purinergic P2X7 receptors, well known for their high Ca^2+^ conductivity, are localized at the membranes of motor nerve terminals and can serve as presynaptic Ca^2+^ inputs [9]. Recently, we showed that the activation of P2X7 receptors by BzATP (30 μM) did not alter evoked end-plate potential (EPP) parameters in wild-type (WT) mice. At the neuromuscular junctions (NMJs) of pannexin-1 knockout (Panx1^−/−^) mice, lacking the pannexin-1-dependent tonic activation of inhibitory presynaptic purinergic receptors, the same activation of presynaptic P2X7 receptors may be accompanied by signaling pathway activation, which includes the activation of calcium-calmodulin-dependent protein kinase type II (CaMKII), the disinhibition of L-type VDCCs and the potentiation of evoked ACh release [10]. However, which of the two modes of activated Ca^2+^ entry, directly via the channels of P2X7 receptors itself or via L-type VDCCs, provides Ca^2+^-dependent facilitation of ACh release and the extent of this facilitation remain unclear. In this context, the aim of the present study was to evaluate the relative contribution of the Ca^2+^ signal generated by Ca^2+^ influx via the channels of P2X7 receptors compared with that of Ca^2+^ influx via L-type VDCCs to the potentiation of evoked ACh release in mouse motor synapses by loading NMJs with Ca^2+^ chelators and through the selective activation of P2X7 receptors and the blockade of L-type VDCCs. Membrane-permeable forms of Ca^2+^ chelators with different Ca^2+^ binding kinetics were used; EGTA-AM was used as a slow Ca^2+^ chelator and BAPTA-AM was used as a fast Ca^2+^ chelator. Both Ca^2+^ chelators were used at the same concentration (50 μM). The use of equimolar concentrations of slow and fast Ca^2+^ chelators allowed us to compare and estimate the dynamics of the effects of various Ca^2+^ signals in nerve terminals and the relative distance from the Ca^2+^ inputs to their targets [11,12]. 

## 2. Results

First, we investigated the effect of intraterminal Ca^2+^ buffering on ACh release in the motor synapses of WT mice. 

**Figure 1:**
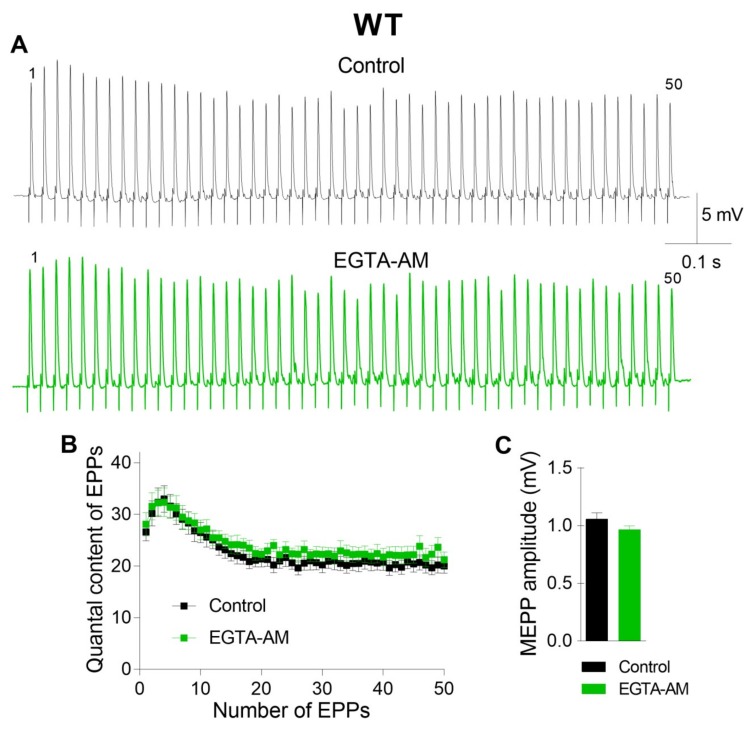
Incubation with the slow Ca^2+^ chelator EGTA-AM (50 µM) did not change evoked neuromuscular transmission at the neuromuscular junctions (NMJs) of wild-type (WT) mice during a short high-frequency train (50 Hz, 1 s). (**A**) Representative recordings of evoked end-plate potentials (EPPs) in the control group (top) and after incubation with EGTA-AM (bottom). (**B**) EPP quantal content in the control group (n = 22) and after incubation with EGTA-AM (n = 15). (**C**) Miniature end-plate potential (MEPP) amplitude in the control group and after incubation with EGTA-AM. The symbols, histograms and error bars represent the mean ± SEM.

The incubation of neuromuscular preparations with EGTA-AM did not change the resting membrane potential (RMP) of muscle fibers (–39.6 ± 1.2 mV in the control (n = 22) and –38.4 ± 1.4 mV after EGTA-AM loading (n = 15, p > 0.05)). Neither spontaneous nor evoked ACh release during short rhythmic trains of stimuli was affected after NMJs were incubated with the slow Ca^2+^ chelator (Figure 1). The miniature end-plate potential (MEPP) amplitude was 1.08 ± 0.06 mV in the control and 0.96 ± 0.03 mV after incubation with EGTA-AM (p > 0.05). The amplitude of the first EPP in the train was 17.41 ± 0.79 mV and 16.67 ± 0.85 mV (p > 0.05) in the control and after incubation with EGTA-AM, respectively. The analysis of EPP quantal content throughout the train revealed that the value of this parameter of ACh release was not influenced by incubation of neuromuscular preparations with the slow Ca^2+^ chelator. Thus, the reduction in presynaptic Ca^2+^ signals induced by the loading of EGTA-AM into nerve terminals did not affect ACh release in the NMJs of WT mice (Figure 1).

**Figure 2:**
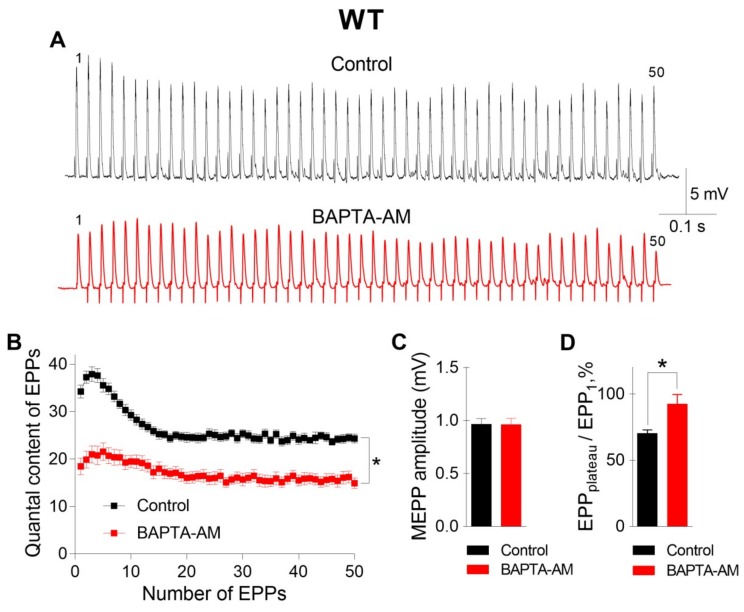
BAPTA-AM induced the downregulation of evoked neuromuscular transmission at the NMJs of WT mice during a short high-frequency train (50 Hz, 1 s). (**A**) Representative recordings of EPPs in the control group (top) and after incubation with BAPTA-AM (bottom). (**B**) Changes in the EPP quantal content in the control group (n = 16) and after incubation with BAPTA-AM (n = 17). (**C**) MEPP amplitude in the control group and after incubation with BAPTA-AM. (**D**) The ratio of the average quantal content of the last ten EPPs in the train to the quantal content of the first EPP (EPP_plateau_/EPP_1_) normalized to EPP_1_ (taken as 100%) in the control group and after incubation with BAPTA-AM. The symbols, histograms and error bars represent the mean ± SEM. * p < 0.05.

The incubation of neuromuscular preparations with 50 μM BAPTA-AM did not lead to any statistically significant changes in MEPP parameters, but, unlike EGTA-AM, BAPTA-AM caused significant decreases in the amplitude and quantal content of EPPs compared to those in the control (Figure 2). In addition, the pattern of the EPP train was transformed in the presence of BAPTA; in addition to a general decrease in the quantal content of EPPs, a significant decrease in the depression phase in the EPP train was observed, while the plateau phase increased in comparison to the first EPP. The ratio of the average quantal content of the last ten EPPs in the train to the quantal content of the first EPP (EPP_plateau_/EPP_1_) increased from 0.70 ± 0.02 in the control (n = 16) to 0.93 ± 0.08 after incubation with BAPTA-AM (n = 17, p < 0.05). This shift in the EPP train pattern reflects a decrease in the probability of ACh release in the presence of BAPTA. As a result, the quantal content of the first EPP in the train was reduced, and there was more pronounced facilitation rather than depression along the rhythmic EPP trains, as has already been shown in the CNS synapses [13]. Thus, only the fast Ca^2+^ chelator BAPTA-AM was able to suppress the Ca^2+^ signals mediated by P/Q-type VDCCs, thus triggering and maintaining the Ca^2+^-dependent evoked release of ACh during EPP trains in mouse motor synapses.

**Figure 3:**
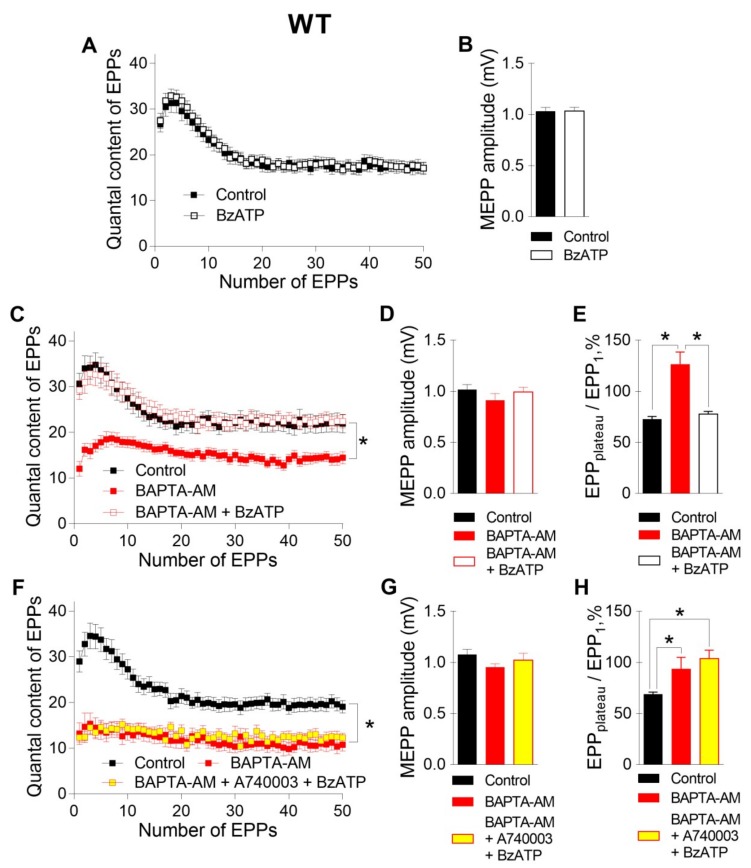
P2X7 receptor-mediated recovery of evoked neuromuscular transmission at the NMJs of WT mice in the presence of a fast Ca^2+^ chelator during a short high-frequency EPP train (50 Hz, 1 s). (**A**) Changes in the EPP quantal content in the control group (n = 24) and during the activation of P2X7 receptors by their agonist BzATP (30 μM) (n = 22). (**B**) MEPP amplitude in the control group and in the presence of BzATP. (**C**) Changes in the EPP quantal content in the control group (n = 16), after incubation with BAPTA-AM (n = 15) and during the subsequent application of BzATP (n = 19). (**D**) MEPP amplitude in the control group, after incubation with BAPTA-AM and during the subsequent application of BzATP. (**E**) The ratio of the average quantal content of the last ten EPPs in the train to the quantal content of the first EPP (EPP_plateau_/EPP_1_) normalized to EPP_1_ (taken as 100%) in the control group, after incubation with BAPTA-AM and during the application of BzATP in the presence of preloaded BAPTA. (**F**) Changes in the EPP quantal content in the control group (n = 22), after incubation with BAPTA-AM (n = 15) and during the subsequent application of BzATP in the presence of P2X7 receptor antagonist A740003 (1 μM) (n = 15). (**G**) MEPP amplitude in the control group, after incubation with BAPTA-AM and during the subsequent application of BzATP in the presence of A740003. (**H**) The ratio of the average quantal content of the last ten EPPs in the train to the quantal content of the first EPP (EPP_plateau_/EPP_1_) normalized to EPP_1_ (taken as 100%) in the control group, after incubation with BAPTA-AM and during subsequent application of BzATP in the presence of A740003. The symbols, histograms and error bars represent the mean ± SEM. * p < 0.05.

Next, we tested the contribution of P2X7 receptors, which might provide specific Ca^2+^ influx to nerve terminals, to the regulation of ACh secretion in NMJs. Like we demonstrated recently [10], the activation of P2X7 receptors by their exogenous agonist BzATP (30 μM) at the NMJs of WT mice did not change either spontaneous or evoked ACh release during rhythmic synaptic activity (Figure 3A,B). However, in the present research, the BzATP-induced stimulation of P2X7 receptors in the presence of preloaded BAPTA caused a dramatic change in the pattern of the EPP trains. Surprisingly, BzATP returned the values of both the amplitude and quantal content of EPPs, which were reduced by incubation with BAPTA-AM in the absence of an effect on spontaneous secretion, to the control level (Figure 3C,D). The EPP_plateau_/EPP_1_ ratio increased from 0.69 ± 0.02 in the control (n = 16) to 1.21 ± 0.13 after incubation with BAPTA-AM (n = 17, p < 0.05) and was then decreased almost to the control value during the application of BzATP in the presence of BAPTA (0.79 ± 0.03, n = 19, p > 0.05) (Figure 3E). Next, we tested whether the BzATP-induced recovery of EPP quantal content is mediated by P2X7 receptors in NMJs of WT mice. In the presence of P2X7 receptor antagonist A740003 (1 μM), BzATP failed to return ACh release, which was decreased in the presence of fast Ca^2+^ chelator, to the control level (Figure 3F-H). Thus, in the presence of BAPTA in nerve terminals, we were able to observe the contribution of P2X7 receptor activity to the regulation of evoked ACh release in the motor synapses of WT mice.

**Figure 4:**
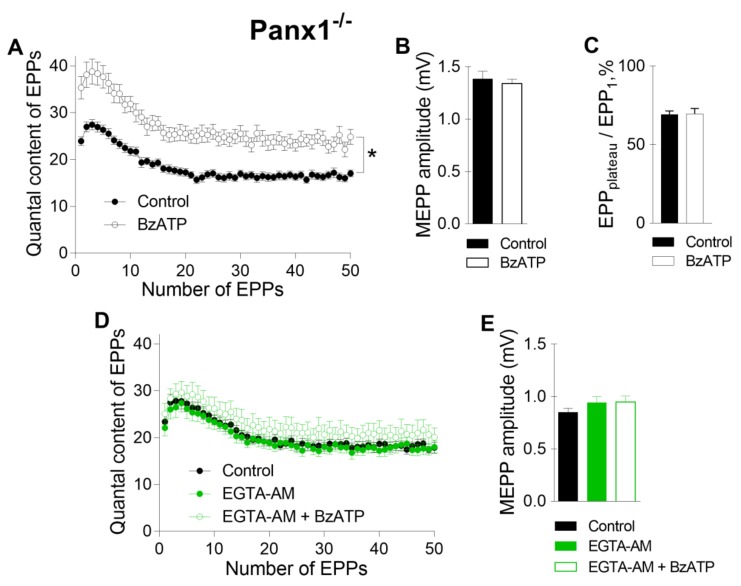
Intraterminal Ca^2+^ buffering with slow Ca2+ chelator prevented P2X7 receptor-mediated potentiation of evoked neuromuscular transmission at the NMJs of pannexin-1 knockout (Panx1^−/−^) mice during a short high-frequency EPP train (50 Hz, 1 s). (**A**) Changes in the EPP quantal content at the NMJs of Panx1^−/−^ mice in the control group (n = 19) and during the activation of P2X7 receptors by their agonist BzATP (30 μM) (n = 15). (**B**) MEPP amplitude in the control group and upon application of BzATP. (**C**) The ratio of the average quantal content of the last ten EPPs in the train to the quantal content of the first EPP (EPP_plateau_/EPP_1_) normalized to EPP_1_ (taken as 100%) in the control group and during the application of BzATP. (**D**) Changes in the EPP quantal content at the NMJs of Panx1^−/−^ mice in the control group (n = 24), after incubation with EGTA-AM (50 µM) (n = 20) and during the application of BzATP in the presence of preloaded EGTA (n = 15). (**E**) MEPP amplitude in the control group, after incubation with EGTA-AM and during the subsequent application of BzATP. The symbols, histograms and error bars represent the mean ± SEM. * p < 0.05.

Further analysis of the specific activity of P2X7 receptors was performed in the NMJs of Panx1^−/−^ mice. Recently, we found that in the motor synapses of Panx1^−/−^ mice but not in those of WT mice, BzATP induces the potentiation of evoked ACh release, which can be prevented by the inhibition of CaMKII or L-type VDCCs [10]. Here, we reproduced the potentiating effect of BzATP in the NMJs of Panx1^−/−^ mice (Figure 4 A–C). We speculated that this effect of BzATP is triggered by the activation of P2X7 receptors followed by the CaMKII-mediated disinhibition of L-type VDCCs, leading to the potentiation of Ca^2+^-dependent ACh release [10]. The role of P2X7 receptor-mediated Ca^2+^ influx as a form of Ca^2+^ entry (in comparison with Ca^2+^ influx through L-type VDCCs) in enhancing ACh secretion in Panx1^−/−^ motor synapses remains unknown. We next tested the ability of fast and slow Ca^2+^ chelators to affect the BzATP-induced potentiation of ACh secretion at the NMJs of Panx1^−/−^ mice. We found that incubation with a slow Ca^2+^ chelator did not change the parameters of spontaneous or evoked release of ACh in the NMJs of Panx1^−/−^ mice, as in the synapses of WT mice. The mean amplitude of MEPPs was 0.83 ± 0.04 mV in the control (n = 24) and 0.94 ± 0.06 mV after incubation with 50 µM EGTA-AM (n = 20, p > 0.05). The mean quantal content of the first EPP in the short rhythmic train was 23.39 ± 1.58 in the control and did not change significantly when EGTA-AM was loaded into nerve terminals (22.09 ± 1.71, p > 0.05). We found that the application of BzATP in the presence of EGTA did not affect the MEPP amplitude, but the ability of BzATP to provoke a significant increase in the quantal content of EPPs was fully prevented by EGTA; the mean value of this parameter for the first EPP in the train was 25.10 ± 2.21 (n = 15, p > 0.05), which was indistinguishable from the control (Figure 4D, E). Thus, the loading of EGTA-AM into motor nerve terminals produced the same effect as that observed upon the blockade of L-type VDCCs: the BzATP-induced potentiation of EPPs was prevented completely in motor synapses [10].

**Figure 5:**
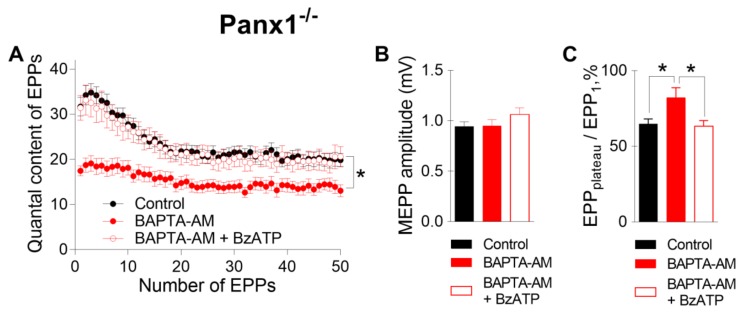
The activation of P2X7 receptors restored the decrease in evoked neuromuscular transmission at the NMJs of Panx1^−/−^ mice induced by incubation with a fast Ca^2+^ chelator. (**A**) Changes in the EPP quantal content in the control group (n = 22), after incubation with BAPTA-AM (50 µM) (n = 17) and during the subsequent activation of P2X7 receptors by their agonist BzATP (30 µM) (n = 15). (**B**) MEPP amplitude in the control group, after incubation with BAPTA-AM and during the subsequent application of BzATP. (**C**) The ratio of the average quantal content of the last ten EPPs in the train to the quantal content of the first EPP (EPP_plateau_/EPP_1_) normalized to EPP_1_ (taken as 100%) in the control group, after incubation with BAPTA-AM and during the application of BzATP in the presence of preloaded BAPTA. The symbols, histograms and error bars represent the mean ± SEM. * p < 0.05.

We found that when BAPTA-AM was used instead of EGTA-AM, the incubation of Panx1^−/−^ NMJs with 50 µM BAPTA-AM did not alter any of the parameters of MEPPs but decreased both the amplitude of EPPs and their quantal content, as in WT mice. (Figure 5). Likewise, a change in the EPP train pattern was observed; the depression of EPPs during the train became less pronounced, and the EPP_plateau_/EPP1 ratio increased from 0.69 ± 0.02 in the control (n = 22) to 1.21 ± 0.13 after incubation with BAPTA-AM (n = 17, p < 0.05) (Figure 5C). The subsequent application of the P2X7 receptor agonist did not affect the spontaneous release of ACh but fully compensated for the inhibitory action of BAPTA on EPP quantal content and restored the pattern of rhythmic EPP train to the control pattern; the EPP_plateau_/EPP_1_ ratio was 0.79 ± 0.03 (n =19) when BzATP was applied to NMJs loaded with BAPTA-AM (Figure 5).

Thus, the loading of BAPTA into motor nerve terminals allowed us to detect the specific contribution of the tonic activation of P2X7 receptors to presynaptic Ca^2+^ signaling associated with BAPTA activity and ACh secretion. These data suggest that the BAPTA-mediated reduction in intraterminal Ca^2+^ signaling followed by the suppression of evoked ACh release can be counterbalanced by the activation of P2X7 receptors and the build-up of additional Ca^2+^ signals provided by Ca^2+^ influx via P2X7 receptor channels and/or L-type VDCCs. Which of the two Ca^2+^ inputs triggered upon the activation of P2X7 receptors is more important and contributes more to the compensation of decreased evoked ACh release induced by the fast Ca^2+^ chelator in motor nerve terminals remains unclear.

**Figure 6:**
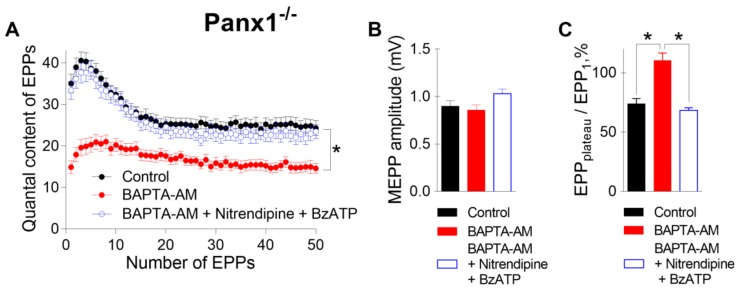
Blocking L-type voltage-dependent Ca^2+^ channels (VDCCs) with nitrendipine (1 µM) failed to prevent the P2X7-mediated recovery of BAPTA-induced depression of evoked neuromuscular transmission at the NMJs of Panx1^−/−^ mice. (**A**) Changes in the EPP quantal content in the control group (n = 24), after incubation with BAPTA-AM (50 µM) (n = 22) and during the subsequent activation of P2X7 receptors by their agonist BzATP (30 µM) in the presence of nitrendipine (n = 21). (**B**) MEPP amplitude in the control group, after incubation with BAPTA-AM and during the subsequent application of BzATP in the presence of nitrendipine. (**C**)The ratio of the average quantal content of the last ten EPPs in the train to the quantal content of the first EPP (EPP_plateau_/EPP_1_) normalized to EPP_1_ (taken as 100%) in the control group, after incubation with BAPTA-AM and during the application of BzATP with nitrendipine in the presence of preloaded BAPTA. The symbols, histograms and error bars represent the mean ± SEM. * p < 0.05.

To answer this question, in the last series of experiments, we incubated neuromuscular preparations from Panx1^−/−^ mice with BAPTA-AM and activated P2X7 receptors in the presence of nitrendipine (1 μM), an inhibitor of L-type VDCCs. Even when L-type VDCCs were blocked, the BzATP-induced activation of P2X7 receptors fully retained the ability to restore the decreased EPP quantal content and the pattern of EPP to the control level despite the presence of BAPTA in the motor nerve terminals (Figure 6). These results suggest that L-type VDCCs do not take part in BzATP-induced compensation of the fast decreases in interterminal Ca^2+^ signaling and evoked ACh release induced by Ca^2+^ chelators. This in turn means that presynaptic Ca^2+^ influx via the channels of P2X7 receptors alone can play a role in maintaining Ca^2+^ homeostasis and counteracting the effect of a fast Ca^2+^ chelator in motor nerve terminals.

Taken together, the obtained data suggest that Ca^2+^ entry via P2X7 receptors may regulate the presynaptic level of free Ca^2+^ when it is reduced by fast Ca^2+^ buffers. This means that under certain conditions, additional presynaptic Ca^2+^ entry via P2X7 receptors may play a special role in maintaining synaptic transmission at NMJs independent of the activation of L-type VDCCs.

## 3. Discussion

In this study, we used two Ca^2+^ chelators with different Ca^2+^ binding kinetics to reveal the role of P2X7 receptors as an additional source of Ca^2+^ entry into motor nerve terminals. We found for the first time that P2X7-mediated Ca^2+^ signaling may have a specific role in presynaptic Ca^2+^ homeostasis independent of its ability to activate CaMKII and L-type VDCCs, as described earlier in our studies [10]. Ca^2+^ buffers are now widely used as an experimental instrument to differentiate the influence of general and additional Ca^2+^ signals on Ca^2+^-dependent neurotransmitter release [11,12].

We established that in the motor synapses of WT mice, a slow Ca^2+^ chelator (EGTA) did not affect the parameters of evoked ACh release, while a fast chelator (BAPTA) significantly reduced both the amplitude and quantal content of the EPPs. This finding is consistent with the generally accepted concept that in mouse motor nerve terminals, fast Ca^2+^ chelators are able to “intercept” Ca^2+^ influx via P/Q-type VDCCs (the main Ca^2+^ input) located in close proximity to synaptic vesicles in active zones. In contrast, slow Ca^2+^ chelators are unable to interfere with this Ca^2+^ signal, which is transient and tightly coupled to vesicular Ca^2+^ sensors and therefore does not affect the quantal content of EPPs in mouse motor terminals [12,14].

Analyzing the roles of additional Ca^2+^ inputs present during the activation of presynaptic P2X7 receptors was our main interest. Recently, we showed that the activation of these receptors by their agonist BzATP does not affect ACh secretion in the motor nerve terminals of WT mice [10]. In the present study, we found for the first time that the BAPTA-induced decrease in EPP quantal content in WT NMJs was reversed back to control values by the application of BzATP. It is well known that P2X7 receptors do not become desensitized [15,16]. Therefore, their tonic activation by BzATP may induce a longer-lasting Ca^2+^ influx than other presynaptic forms of Ca^2+^ entry and cause partial saturation of preloaded Ca^2+^ chelators. Thus, our data suggest that Ca^2+^ input via activated P2X7 receptors can augment the presynaptic Ca^2+^ concentration and thereby decrease the buffering capacity of Ca^2+^ chelators, which is manifested as the restoration of the BAPTA-induced depression of neuromuscular transmission back to control levels.

We previously showed that the supply of endogenous ATP/adenosine to the synaptic terminals via pannexin-1 hemichannels and the activation of various presynaptic purinoreceptors is abolished at the NMJs of Panx1^−/−^ mice. Pannexin-1 knockout is primarily followed by a lack of activation of metabotropic A1- and P2Y13 receptors, which inhibit (silence) L-type VDCCs and their coupling to ACh secretion [10,17]. As a result, in the NMJs of Panx1^−/−^ mice, the activation of P2X7 receptor function by BzATP becomes more pronounced and is accompanied by the activation of presynaptic CaMKII, leading to the potentiation of ACh secretion due to the activation of L-type VDCCs, which can be blocked by nitrendipine [10]. However, the role of Ca^2+^ that enters the nerve terminal via activated P2X7 receptors alone remains unclear. Can this additional Ca^2+^ signal play a distinct role in neurotransmitter release independent of and/or in parallel with the role of simultaneously activated L-type VDCCs? To answer this question, we studied the effects of Ca^2+^ chelators and their influence on different Ca^2+^ inputs in nerve terminals and ACh secretion during the activation of P2X7 receptors in the NMJs of Panx1^−/−^ mice.

We found for the first time that the incubation of Panx1^−/−^ NMJs with Ca^2+^ chelators induced different changes in evoked ACh release, as in the motor synapses of WT mice; BAPTA reduced both the amplitude and the quantal content of EPPs and changed the pattern of EPP train, while EGTA failed to do so. Hence, it is reasonable to suggest that in Panx1^−/−^ mice, the triggering and regulation of evoked ACh release occurs via the same form of Ca^2+^ entry as in the motor synapses of WT mice: fast P/Q-type VDCCs.

Our previous data demonstrated that in Panx1^−/−^ mice, the activation of P2X7 receptors causes a uniform increase in the amplitude and quantal content of EPPs in short EPP trains by an average of 30% relative to the control. This P2X7-mediated effect can be prevented by nitrendipine, suggesting the involvement of L-type VDCCs in the regulation of ACh release as the final target of P2X7 receptor activity [10].

In the present study, we found that loading EGTA-AM into nerve terminals, like nitrendipine application, fully prevented the BzATP-induced facilitation of evoked ACh release. In the presence of BAPTA, BzATP also lost its ability to enhance the EPP quantal content above the control level; the activation of P2X7 receptors returned the decreased quantal content of EPPs to the control level but not beyond this level. Of special interest is the fact that nitrendipine (1 μM) was unable to prevent this BzATP-induced potentiation of EPPs to the control level in the presence of BAPTA.

The most likely reason for the ability of BzATP to enhance evoked ACh release to the control level despite the presence of BAPTA is the partial saturation of the BAPTA buffering capacity by Ca^2+^ entering via the channels of P2X7 receptors. As a result, the buffering capacity of BAPTA-AM becomes insufficient to effectively reduce the Ca^2+^ signal triggered by P/Q-type VDCC activation, and therefore, both the amplitude and quantal content of EPPs quickly return to the control values upon the stimulation of P2X7 receptors in the presence of the fast Ca^2+^ chelator. Similar partial saturation of BAPTA and EGTA as a result of P2X7-mediated influx of Ca^2+^ has been observed in other cells [18,19].

**Figure 7:**
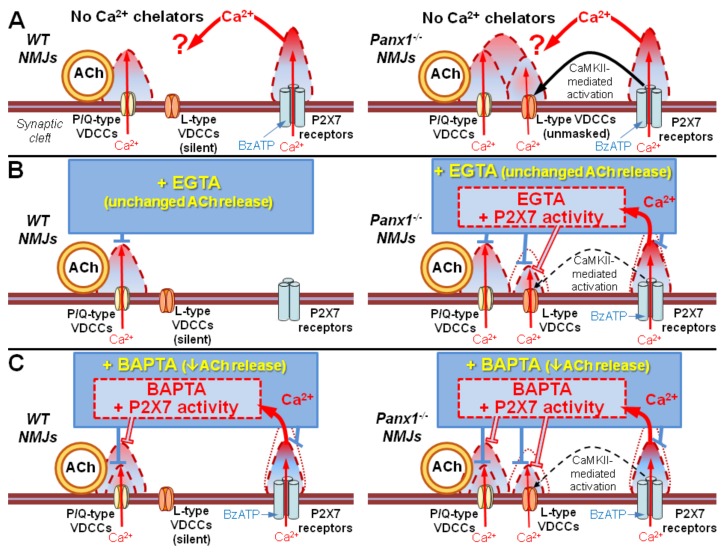
Suggested contributions of various presynaptic Ca^2+^ inputs, interacting with slow or fast Ca^2+^ chelators, to evoked synaptic transmission in mouse NMJs. (**A**) Without Ca^2+^ chelators, activation of P2X7 receptors facilitates ACh release only in NMJs of Panx1^−/−^ mice due to activation of CAMKII and L-type VDCCs [10]. (**B**) In NMJs of WT mice, where L-type VDCCs are inactive, EGTA failed to change ACh release due to an inability of the slow Ca^2+^ chelator to affect Ca^2+^ entry via P/Q-type VDCCs. Activation of P2X7 receptors in the presence of EGTA would not induce any more changes since the stimulation of P2X7 receptors fails to disinhibit L-type VDCCs in NMJs of WT mice [10]. In NMJs of Panx1^−/−^ mice, P2X7 receptor-mediated Ca^2+^ entry partially decreases EGTA buffering capacity, which is still sufficient to disrupt coupling of Ca^2+^ entry via L-type VDCCs to ACh release. (**C**) Both in NMJs of WT and Panx1^−/−^ mice, Ca^2+^ entry via P2X7 receptors partially decreases BAPTA buffering capacity, leading to restoration of P/Q-type VDCC-triggered ACh release. In NMJs of Panx1^−/−^ mice, buffering capacity of BAPTA, even diminished by Ca2+ entry via P2X7 receptors, is still sufficient to downregulate Ca^2+^ entry via L-type VDCCs, thus preventing the facilitation of ACh release. Triangles with dashed borders represent the proposed changes in the presynaptic Ca^2+^ domains, which correspond to the activity of distinct Ca^2+^ inputs during synaptic activity in mouse NMJs.

It is worth noting that despite the probable partial saturation due to the influx of Ca^2+^ via P2X7 receptors, both fast and slow Ca^2+^ chelators were able to equally and effectively prevent the potentiation of EPP quantal content above the control level, which was observed in the absence of incubation with Ca^2+^ chelators upon the stimulation of P2X7 receptors followed by the upregulation of Ca^2+^ input via L-type VDCCs in the NMJs of Panx1^−/−^ mice. This means that both Ca^2+^ chelators, even when partially saturated due to P2X7 receptor activity, retained sufficient buffering capacity to effectively “quench” the Ca^2+^ signal generated by L-type VDCCs, thereby preventing its potentiating effect on the evoked quantal release of ACh. Apparently, this is due to the short-term and relatively weak Ca^2+^ signals generated by opened L-type VDCCs in motor nerve terminals compared to the long-lasting Ca^2+^ influx via the channels of tonically active P2X7 receptors [20]. Overall, the proposed interactions between Ca^2+^ chelators and the activity of P2X7 receptors in regulation of Ca^2+^-dependent ACh release in mouse NMJs are depicted in Figure 7.

## 4. Materials and Methods 

### 4.1. Animals and Neuromuscular Preparations 

Electrophysiological experiments were performed on isolated neuromuscular preparations of the hemidiaphragms (m. diaphragm – n. phrenicus) of age-matched adult C57BL/6 mice (7–8 weeks old, weighing 25–30 g) (wild-type, WT) or pannexin-1 knockout (Panx1^−/−^) mice of either sex. The characteristics of Panx1^−/−^ mice were described previously [21]. Both animal handling and experimental procedures were carried out in full compliance with the EC guidelines (Directive 86/609/EEC on the protection of animals used for experimental and scientific purposes). Mice were housed with free access to food and water under standard humidity and temperature conditions with a 12-h light/dark cycle. The Bioethics Committee of the MSU Biological Department approved the experimental protocol (number 2019-06-27-102-0). The mice were sacrificed by quick decapitation. A total of 30 animals were used in the study.

### 4.2. Electrophysiology

All experiments were performed at room temperature (20–22 °C). The left hemidiaphragm with the phrenic nerve attached was dissected quickly and stretched in a 3-mL Sylgard-coated chamber perfused (0.4–0.5 mL/min) with oxygenated (95% O_2_, 5% CO_2_) Liley solution containing 135 mM NaCl, 4 mM KCl, 0.9 mM NaH_2_PO_4_, 2 mM CaCl_2_, 1 mM MgCl_2_, 16.3 mM NaHCO_3_ and 11 mM glucose. To prevent contraction at the time of recordings of synaptic activity evoked by nerve stimulation, the muscle fibers of the neuromuscular preparations were transversely cut. This procedure allowed both spontaneous MEPPs and multiquantal EPPs to be recorded from the same synapse [22]. After the muscle fibers were cut, the neuromuscular preparations were washed thoroughly with a large volume of Liley solution (more than 150 mL) for more than 1 h to prevent the blockage of action potential conduction. As a result, the recorded value of the RMP was lower in cut fibers (less than –50 mV) than in intact fibers. Glass intracellular microelectrodes filled with 2.5 M KCl (10–20 MΩ of tip resistance) and connected to a Neuroprobe Amplifier, model 1600 (A-M Systems, Sequim, WA, USA) were used to record MEPPs and EPPs. The signals were digitized using an analog–digital converter E-154 (L-Card, Moscow, Russia) with a PowerGraph 6.0 interface. The phrenic nerve was stimulated by short high-frequency (50 Hz, 1 s) trains of suprathreshold pulses (the duration of each pulse was 0.08–0.1 ms) to study evoked synaptic activity. An interval of no less than 4 min was allowed between nerve stimulations to avoid synaptic fatigue and alterations in EPP patterns not related to drug application. MEPPs were recorded for 100 s prior to nerve stimulation in each synapse (the mean value of the MEPP amplitudes recorded within this period was used to calculate the quantal content of EPPs). In the control, MEPPs and rhythmically evoked EPPs from 5–8 synapses were recorded, after which the studied substances were added to the perfusion solution in a certain order. In each series of experiments, at least three neuromuscular preparations were used, and the synaptic activity of at least 15 separate synapses was recorded for each group.

### 4.3. Loading of Motor Nerve Terminals with Membrane-Permeable Ca^2+^ Chelators

After control recordings, the neuromuscular preparations were incubated for 60 minutes in a calcium-free solution of 135 mM NaCl, 4 mM KCl, 0.9 mM NaH_2_PO_4_, 3.2 mM MgCl_2_, 16.3 mM NaHCO_3_ and 11 mM glucose. This solution also contained an AM forms of a Ca^2+^ chelator (BAPTA-AM or EGTA-AM, 50 μM). Next, the neuromuscular preparations were washed for 15 min with the calcium-free solution without Ca^2+^ chelators. Then, the washing solution was replaced with Liley solution with a normal concentration of Ca^2+^ (2 mM), and the neuromuscular preparations were washed with this solution for 15 minutes, after which recordings of synaptic activity were initiated.

### 4.4. Data Processing and Statistical Analysis

Primary analysis of electrophysiological data was performed using MiniAnalysis software (Synaptosoft, Decatur, GA, USA). The values of the RMP of muscle fibers and the amplitude and time course of MEPPs and EPPs in short rhythmic trains were evaluated. To mitigate changes in the driving force caused by a shift in voltage upon RMP changes, the amplitudes of the MEPPs and EPPs were standardized to a –50 mV membrane potential [23]. The EPP quantal content was calculated as the ratio of the mean standardized EPP amplitude corrected for nonlinear summation to the mean standardized MEPP amplitude [24]. Statistical analysis was performed using Prism 6.0 software (GraphPad Software, San Diego, CA, USA). In the text and figures, all of the data except the original recordings are presented as the means ± standard error of the means, with the n value representing the number of synapses in the group. The deviation of the distribution from normal for all parameters was evaluated using the D’Agostino–Pearson test. To assess the RMP, the MEPP amplitude and the ratio of the EPP quantal content at plateau level of the EPP train (mean of the last 10 EPPs) to the quantal content value of the first EPP in the train in the case of normally distributed values, we used Student’s *t*-test or one-way ANOVA. In the case of non-normal distribution, the Mann–Whitney test and the Kruskal–Wallis test were used. To analyze the amplitude and quantal content of EPPs, two-way ANOVA followed by the post hoc Bonferroni correction (when comparing two groups) or Tukey’s test (when comparing three groups) was used. Values of p < 0.05 were considered significant for all analyses.

### 4.5. Drugs

The fast Ca^2+^ chelator (BAPTA-AM), the slow Ca^2+^ chelator (EGTA-AM) and P2X7 receptor antagonist A740003 were purchased from Tocris/Bio-Techne (Minneapolis, MN, USA) and Calbiochem Biochemicals (Nottingham, Great Britain), respectively. The P2X7 receptor agonist BzATP was purchased from Sigma-Aldrich (St. Louis, MO, USA). The L-type VDCC inhibitor nitrendipine was obtained from Biomol GmbH (Hamburg, Germany). Stock solutions of Ca^2+^ chelators, A740003 and nitrendipine were prepared using DMSO (Helicon, Moscow, Russia) as the solvent. BzATP was dissolved in deionized water. The final concentration of DMSO in the working solution did not exceed 0.01% (v/v) and did not affect spontaneous or evoked activity in mouse NMJs.

## 5. Conclusions

In summary, it was revealed for the first time that Ca^2+^ entry via the channels of P2X7 receptors can act as an additional presynaptic source of Ca^2+^, which can partially compensate for deficiencies in Ca^2+^ signaling induced by Ca^2+^ chelators in motor nerve terminals. This Ca^2+^ input, which is mediated by tonically active P2X7 receptors, can partially downregulate the buffering capacity of Ca^2+^ chelators and recover the fast chelator-induced decrease in Ca^2+^ signals entering via P/Q-type VDCCs, triggering direct ACh secretion. This means that P2X7-induced Ca^2+^ input alone may support presynaptic Ca^2+^ homeostasis. Moreover, Ca^2+^ entry through P2X7 receptors may indirectly contribute to the enhancement of evoked ACh release via triggering the activation of CaMKII followed by upregulation of L-type VDCCs [10]. According to our present and previous studies, we conclude that P2X7-mediated Ca^2+^ entry is primarily intended to trigger the activation of calcium-dependent enzymes and ultimately upregulate L-type VDCCs, which in turn can potentiate Ca^2+^-dependent ACh release in mouse motor synapses. The variety of effects revealed by the activation of P2X7 receptors is of practical interest for understanding their specific role in the activity of motor synapses under certain normal and pathological conditions.

## Abbreviations

ACh	Acetylcholine
VDCCs	Voltage-dependent calcium channels
WT	Wild-type
Panx1^−/−^	Pannexin-1 knockout
MEPP	Miniature end-plate potential
EPP	Evoked end-plate potential
EGTA-AM	ethylene glycol-bis(2-aminoethylether)-N,N,N’,N’-tetraacetic acid acetoxymethyl ester
BAPTA-AM	1,2-bis(2-aminophenoxy)ethane-N,N,N’,N’-tetraacetic acid tetrakis acetoxymethyl ester
BzATP	2′(3′)-O-(4-benzoylbenzoyl)-adenosine 5′-triphosphate triethylammonium salt
A740003	*N*-[1-[[(Cyanoamino)(5-quinolinylamino)methylene]amino]-2,2-dimethylpropyl]-3,4- dimethoxybenzeneacetamide
CaMKII	Calcium-calmodulin-dependent protein kinase type II
RMP	Resting membrane potential
